# Supplementary material for: Promoting social distancing in a pandemic: Beyond good intentions
Source: PLoS One. 2021 Dec 2;16(12):e0260457. doi: 10.1371/journal.pone.0260457 (PMC8638903; doi:10.1371/journal.pone.0260457)
Supplement: S1 Appendix — (PDF) [file pone.0260457.s001.pdf]

Supporting information

Appendix A. Test of Balance In Table 4, we test balance across treatments in the sociodemographic characteristics of respondents provided by Statistics Denmark. For each covariate, we report the difference between its mean in the different treatment groups and the control mean (with standard errors in parentheses). The results show that covariates are balanced across treatment groups.

Table 4. Balance of covariates across treatments.

Variable	Categories	Treatments					Control mean	N
		Personal	Family	Others	Country	Generic		
Gender	M	0.039	0.013	-0.019	0.022	0.032	0.423	5,310
		(-0.026)	(-0.026)	(-0.026)	(-0.026)	(-0.030)	(-0.021)	
	F	-0.039	-0.0132	0.0192	-0.0223	-0.0324	0.577	5,310
		(-0.026)	(-0.026)	(-0.026)	(-0.026)	(-0.030)	(-0.021)	
Age	18-29	0.00276	0.00437	0.0132	0.0166	-0.0111	0.144	5,310
		(-0.018)	(-0.019)	(-0.019)	(-0.019)	(-0.021)	(-0.015)	
	30-39	0.00342	0.00579	-0.0067	-0.0079	0.0119	0.138	5,310
		(-0.018)	(-0.018)	(-0.018)	(-0.018)	(-0.022)	(-0.015)	
	40-49	-0.0168	-0.017	-0.0236	-0.0026	-0.0253	0.205	5,310
		(-0.021)	(-0.021)	(-0.021)	(-0.021)	(-0.024)	(-0.017)	
	50-59	-0.0041	0.0125	0.0108	0.00833	0.0311	0.248	5,310
		(-0.023)	(-0.023)	(-0.023)	(-0.023)	(-0.027)	(-0.018)	
	60-69	0.0147	-0.0057	0.00637	-0.0144	-0.0066	0.264	5,310
		(-0.023)	(-0.023)	(-0.023)	(-0.023)	(-0.027)	(-0.019)	
Region	Nordjylland	-0.0164	-0.024	-0.0221	-0.0179	0.00645	0.121	5,310
		(-0.017)	(-0.017)	(-0.017)	(-0.017)	(-0.020)	(-0.014)	
	Midtjylland	-0.0243	-0.0349	-0.0306	-0.0266	-0.0167	0.261	5,310
		(-0.023)	(-0.023)	(-0.023)	(-0.023)	(-0.027)	(-0.019)	
	Syddanmark	0.00812	0.0523**	0.0206	0.0146	0.0185	0.192	5,310
		(-0.021)	(-0.022)	(-0.021)	(-0.021)	(-0.025)	(-0.017)	
	Hovedstaden	0.0217	0.00986	0.0336	0.0133	-0.04	0.308	5,310
		(-0.024)	(-0.025)	(-0.024)	(-0.024)	(-0.028)	(-0.020)	
	Sjælland	0.0109	-0.0033	-0.0014	0.0166	0.0317	0.119	5,310
		(-0.017)	(-0.017)	(-0.017)	(-0.017)	(-0.021)	(-0.014)	
Highest completed education	Elementary school	-0.0112	-0.0349*	-0.0057	0.00717	0.00461	0.167	5,310
		(-0.019)	(-0.019)	(-0.019)	(-0.020)	(-0.023)	(-0.016)	
	Youth Education	0.00224	0.00952	0.0186	0.00797	0.0432	0.388	5,310
		(-0.026)	(-0.026)	(-0.025)	(-0.026)	(-0.030)	(-0.021)	
	Short + Medium Long Ex.	-0.0056	0.0202	-0.0153	0.0205	-0.0331	0.273	5,310
		(-0.023)	(-0.024)	(-0.023)	(-0.024)	(-0.027)	(-0.019)	
	Long Higher education	0.0146	0.00526	0.00242	-0.0356*	-0.0146	0.171	5,310
		(-0.020)	(-0.020)	(-0.020)	(-0.019)	(-0.023)	(-0.016)	
Equivalent disposable income for the family	<150	-0.0175	-0.0074	-0.0003	-0.0045	-0.0199	0.155	5,310
		(-0.019)	(-0.019)	(-0.019)	(-0.019)	(-0.022)	(-0.015)	
	150-250	-0.0293	-0.0255	-0.0081	0.00487	-0.0093	0.255	5,310
		(-0.023)	(-0.023)	(-0.023)	(-0.023)	(-0.027)	(-0.019)	
	250-350	0.0265	0.0311	0.00189	0.0046	0.0723**	0.273	5,310
		(-0.024)	(-0.024)	(-0.023)	(-0.023)	(-0.028)	(-0.019)	
	>350	0.0203	0.00186	0.00656	-0.005	-0.0431	0.317	5,310
		(-0.025)	(-0.025)	(-0.024)	(-0.024)	(-0.028)	(-0.020)	

Notes: For each covariate we test the difference in means between the control group and the treatment groups. Each cell shows the difference with standard errors in parentheses. Confidence: *** p<0.01, ** p<0.05, * p<0.10.

Appendix B. Treatments This section shows the reminders as they appeared to respondents. For each reminder, we indicate the main focus of the framing ("you", "family", "others", "country") and whether the message was framed in terms of the risks from not complying ("loss") or the benefits from complying ("gain") with the recommendation to stay home.

Appendix C. Questionnaires This section reports the questions that form part of the two questionnaires we administered. Respondents completed the first questionnaire immediately after reading the reminder. They received and completed the second questionnaire in the following days (no earlier than two days after completing the first questionnaire to prevent inconsistent answers).

Table 5. Treatments

FRAME<br>(Consequences for...)	DOMAIN<br>(Loss Gain)	MESSAGE
CONTROL GROUP		NO REMINDER
PERSONAL	Loss	IF YOU GO OUTSIDE AND BECOME INFECTED, **YOU MAY GET** VERY SERIOUS RESPIRATORY PROBLEMS<br>STAY HOME AS MUCH AS POSSIBLE
	Gain	IF YOU STAY HOME, **YOU PROTECT YOURSELF** FROM THE RISK OF GETTING VERY SERIOUS RESPIRATORY PROBLEMS<br>STAY HOME AS MUCH AS POSSIBLE
FAMILY	Loss	**THINK OF YOUR LOVED ONES**<br>IF YOU GO OUTSIDE AND BECOME INFECTED, **YOU MAY INFECT THEM,** AND THEY MAY GET VERY SERIOUS RESPIRATORY PROBLEMS<br>STAY HOME AS MUCH AS POSSIBLE
	Gain	**THINK OF YOUR LOVED ONES**<br>IF YOU STAY HOME, **YOU PROTECT THEM** FROM THE RISK OF GETTING VERY SERIOUS RESPIRATORY PROBLEMS<br>STAY HOME AS MUCH AS POSSIBLE

OTHERS	Loss	IF YOU GO OUTSIDE AND BECOME INFECTED, **YOU MAY INFECT OTHERS,** WHO MAY GET VERY SERIOUS RESPIRATORY PROBLEMS<br>STAY HOME AS MUCH AS POSSIBLE
	Gain	IF YOU STAY HOME, **YOU PROTECT OTHERS** FROM THE RISK OF GETTING VERY SERIOUS RESPIRATORY PROBLEMS<br>STAY HOME AS MUCH AS POSSIBLE
COUNTRY	Loss	IF YOU GO OUTSIDE AND BECOME INFECTED, **YOU MAY CONTRIBUTE TO AN OVERLOADING OF THE DANISH HEALTH CARE SYSTEM**<br>STAY HOME AS MUCH AS POSSIBLE
	Gain	IF YOU STAY HOME, **YOU REDUCE THE RISK OF AN OVERLOADING OF THE DANISH HEALTH CARE SYSTEM**<br>STAY HOME AS MUCH AS POSSIBLE
GENERIC WARNING		STAY HOME AS MUCH AS POSSIBLE

Table 6 reports the English translation of all the questions in the first questionnaire. Table 7 reports the English translation of all the questions in the second questionnaire.

Table 6. First questionnaire.

	QUESTION	OPTIONS
Q1	On a scale from 1 to 5, how worried do you feel at the moment?	1-5
Q2	On a scale from 1 to 5, how sad do you feel at the moment?	1-5
Q3	How do you think your health is overall?	- Excellent<br>- Very good<br>- Good<br>- Not very good<br>- Bad<br>- Do not know
Q4	For how long do you think you will go out of your house tomorrow?<br><br>Please give your answer in minutes and/ or hours<br>(If you are spending time in your own garden, it is seen as staying home, so you should not include this time in your answer)	Hours and Minutes
Q5	What is the maximum distance from home you are going to reach tomorrow?<br><br>Please give your answer in meters and/or kilometers<br>(If you are spending time in your own garden, it is seen as staying and you should answer "0")	Kilometers and Meters
Q6	For how long do you think, on average, other people in Denmark will go out tomorrow, on average?<br><br>Please give your answer in minutes and/ or hours	Hours and Minutes
Q7	On a scale of 1 to 100%, how likely do you think it is that you will be infected with the Coronavirus?	1-100
Q8	To what extent do you trust the Danish government to take care of the citizens of the country in connection with the Corona situation?	- Strongly distrust<br>- Somewhat distrust<br>- Neither trust not distrust<br>- Somewhat trust<br>- Strongly trust<br>- Refusing to answer<br>- Do not know

Table 7. Follow-up questionnaire.

	QUESTION	OPTIONS
Q1	On a scale from 1 to 5, where 1 means very little and 5 means very much, how angry do you feel at the moment?	1-5
Q2	On a scale from 1 to 5, where 1 means very little and 5 means very much, how worried do you feel at the moment?	1-5
Q3	On a scale from 1 to 5, where 1 means very little and 5 means very much, how sad do you feel at the moment?	1-5
Q4	For how long did you leave your home yesterday?<br><br>(If you are spending time in your own garden, it is seen as staying home, so you should not include this time in your answer)	Hours and Minutes
Q5	What was the maximum distance from home you reached yesterday?	Kilometers and Meters
Q6	What were the reasons for you to leave your home yesterday (check all that apply)?	- Work<br>- Purchase<br>- Physical activity (e.g., walking, running, cycling)<br>- Medical treatment (e.g., in hospital or at own doctor)<br>- Drugstore<br>- Visiting / caring for relatives<br>- Ventilate a pet<br>- Meet with friends or family<br>- Other:
Q7	Did someone else who lives with you go out yesterday?<br><br>(If the individual was only in his/her own garden, it is seen as staying hope and you are asked to answer "no")	- Yes<br>- No<br>- Refusing to answer<br>- Do not know
Q8	How much do you trust the Danish government to take care of its citizens?	- Strongly distrust<br>- Somewhat distrust<br>- Neither trust not distrust<br>- Somewhat trust<br>- Strongly trust<br>- Refusing to answer<br>- Do not know
Q9	On a scale from 0-100 how important do you think it is that everyone stays at home in Denmark?	0-100
Q10	On a scale from 0 - 100 how important do you think others believe it is to stay at home in Denmark?	0-100
Q11	How effective do you think the social distancing measures are in slowing down the spread of the coronavirus?	- Very effective<br>- Effective<br>- Neither effective nor ineffective<br>- Not effective<br>- Not effective at all<br>- Do not know
Q12	Which of the following consequences of the epidemic are you most concerned about?	- Health system overload<br>- Economic consequences of the shutdown for Denmark<br>- Financial consequences of the shutdown for my family<br>- The effect of isolation on my well-being<br>- The impact on my social life and lifestyle<br>- Other things
Q13	How strongly do you agree with the following statement?<br><br>"The healthcare system will be overloaded by the COVID19 epidemic".	- Strongly agree<br>- Agree<br>- Neither agree or disagree<br>- Disagree<br>- Strongly disagree<br>- Refusing to answer<br>- Do not know
Q14	How many people in Denmark do you think will be infected (tested positive) with Coronavirus in a month?	0-...

Appendix D. Robustness Checks

Appendix E. Timeline and Mobility Trends In this section, we first report the timeline of the most salient events that occurred during the most critical period of the first pandemic wave in Denmark, up to the end of our study period (Fig 4).

Fig 4. Timeline of relevant events related to the COVID-19 pandemic in Denmark. *Notes*: The figure shows that our intervention took place at a critical moment during the first wave of the pandemic, when maximum attention was placed on social distancing.

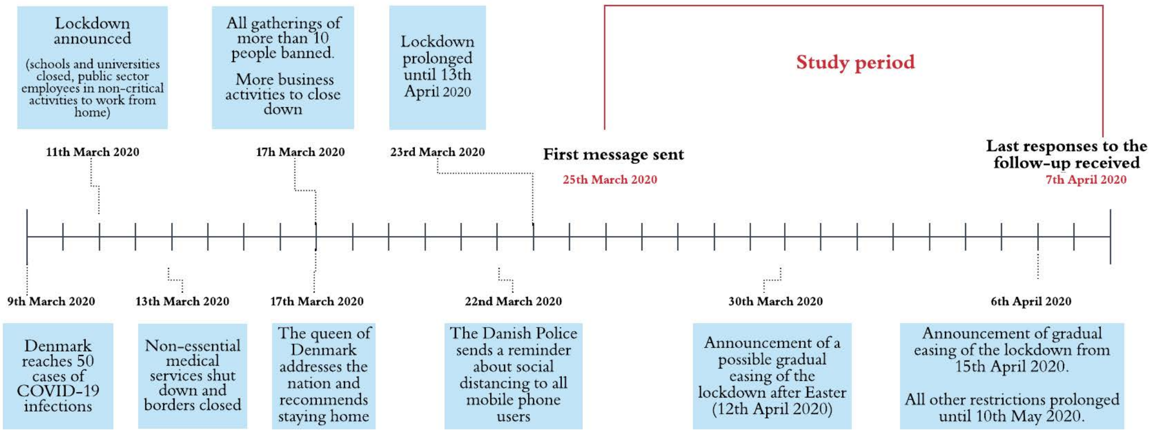

Next, we show that our data on respondents' mobility closely tracks a widely used measure of mobility based on Apple Inc.'s data from mobile-phone users in Denmark (Fig 5).

Fig 5. Mobility trends in Demark based on mobile data vs our survey. *Notes*: The figure shows data on how much people travel in Denmark over the period analysed. It compares data from a question on the distance travelled by the respondents in our survey with data from Apple Inc. obtained from routing requests made to Apple web mapping service (Link). For comparability, we plot percentage changes relative to the first date in the period. We exclude extreme values (top 1% largest distances) and individuals who travelled < 3km, since the respondents who are most likely to use a web mapping service (and to be comparable to those submitting routing requests to Apple) are the ones traveling outside their own neighborhood. If we relax this restriction, the results are similar, albeit less precise.

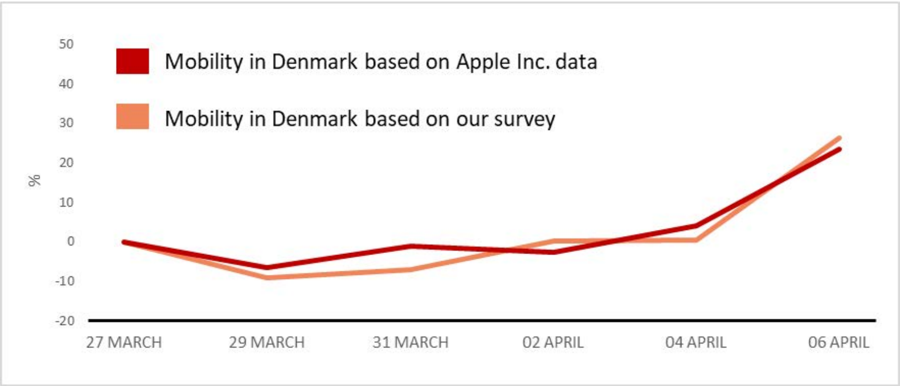

Appendix F. Attrition In this section, we test whether the probability that a respondent drops out of the sample between the first survey and the follow-up survey correlates with assignment to treatment. The raw data in Table 10 show that attrition is strongly balanced across treatment groups.

As a formal test, we regress the probability of dropping out of the sample on being in each of the treatment groups and we find no relationship (Table 11). When we run the same analysis for the sub-treatments, we reach the same conclusion (only the sub-treatment "Others" in the gain domain is associated with a lower probability of dropping out of the sample, but the result is only significant at the 10% level).

Table 8. Robustness checks.

	Non-matching days included		Exclude subjects who spend 24h out of home		Excluded subjects who answered the follow-up later than 1 w after the first survey		First week only		Aggregate treatments	
	(1)	(2)	(3)	(4)	(5)	(6)	(7)	(8)	(9)	(10)
VARIABLES	Intention	Action	Intention	Action	Intention	Action	Intention	Action	Intention	Action
You loss	0.031*	0.0214	0.0441**	0.0281	0.0440**	0.0264	0.0254	0.0251		
	(0.018)	(0.0207)	(0.0197)	(0.0224)	(0.0197)	(0.0225)	(0.0226)	(0.0256)		
You gain	0.018	0.0106	0.0441**	0.0272	0.0414**	0.0304	0.0417*	0.0436*		
	(0.017)	(0.0201)	(0.0195)	(0.0221)	(0.0194)	(0.0223)	(0.0231)	(0.0259)		
Family loss	0.031*	0.0155	0.0462**	0.0236	0.0530***	0.0278	0.0575**	0.0315		
	(0.018)	(0.0205)	(0.0199)	(0.0223)	(0.0203)	(0.0228)	(0.0243)	(0.0261)		
Family gain	0.020	0.00403	0.0367*	0.0210	0.0416**	0.0199	0.0323	0.0325		
	(0.018)	(0.0204)	(0.0198)	(0.0225)	(0.0200)	(0.0226)	(0.0230)	(0.0260)		
Others loss	0.006	0.00507	0.00265	-0.000157	0.00599	-0.00783	0.00415	-0.0122		
	(0.017)	(0.0201)	(0.0182)	(0.0215)	(0.0183)	(0.0215)	(0.0218)	(0.0242)		
Others gain	0.017	0.00416	0.0192	0.0225	0.0200	0.0260	0.0132	0.0307		
	(0.017)	(0.0196)	(0.0183)	(0.0216)	(0.0184)	(0.0219)	(0.0214)	(0.0249)		
Country loss	0.019	0.0111	0.0272	0.0190	0.0292	0.0193	0.0180	0.00584		
	(0.017)	(0.0199)	(0.0187)	(0.0216)	(0.0187)	(0.0218)	(0.0219)	(0.0245)		
Country gain	0.027	0.0375*	0.0233	0.0448**	0.0221	0.0393*	0.00874	0.0311		
	(0.018)	(0.0209)	(0.0190)	(0.0228)	(0.0190)	(0.0229)	(0.0221)	(0.0260)		
You and Family									0.0438***	0.0253
									(0.0155)	(0.0178)
Others and Country									0.0183	0.0215
									(0.0155)	(0.0177)
Generic	0.017	0.00233	0.0238	0.0353	0.0255	0.0349	0.000467	0.0419	0.0249	0.0321
	(0.017)	(0.0199)	(0.0191)	(0.0225)	(0.0191)	(0.0226)	(0.0214)	(0.0259)	(0.0199)	(0.0228)
Controls	Yes	Yes	Yes	Yes	Yes	Yes	Yes	Yes	Yes	Yes
Observations	6,674	6,674	5,249	5,249	5,160	5,160	3,843	3,843	5,310	5,310

Notes: Intentions refer to the day after the first interview, actions refer to the day before the follow-up interview. Controls include the following balancing covariates (used at the randomisation stage): gender, age, region, education, and household disposable income per capita. Robust standard errors in parentheses. Confidence: *** p<0.01, ** p<0.05, * p<0.10.

Table 9. Effect of treatments and sub-treatments on staying home controlling for day of week.

	(1)	(2)	(3)	(4)
VARIABLES	Intention	Action	Intention	Action
You	0.044***	0.0287		
	(0.016)	(0.0188)		
Family	0.043***	0.0217		
	(0.016)	(0.0189)		
Others	0.011	0.0135		
	(0.016)	(0.0185)		
Country	0.025	0.0297		
	(0.016)	(0.0188)		
You loss			0.0448**	0.0273
			(0.0196)	(0.0223)
You gain			0.0428**	0.0300
			(0.0193)	(0.0221)
Family loss			0.0470**	0.0250
			(0.0198)	(0.0223)
Family gain			0.0398**	0.0183
			(0.0197)	(0.0223)
Others loss			0.00412	-0.000498
			(0.0181)	(0.0214)
Others gain			0.0182	0.0261
			(0.0181)	(0.0216)
Country loss			0.0268	0.0170
			(0.0185)	(0.0215)
Country gain			0.0236	0.0436*
			(0.0189)	(0.0227)
Generic	0.025	0.0321	0.0248	0.0321
	(0.019)	(0.0223)	(0.0190)	(0.0224)
Controls	Yes	Yes	Yes	Yes
Observations	5,310	5,310	5,310	5,310

Table 10. Number of respondents and attrition rate.

GROUP	DOMAIN	Completed first survey	Completed follow-up survey	Attrition rate
Group 0		1,285	692	46%
Group 1		1,268	674	47%
Group 2	You	2,472	1,311	47%
Group 3	Family	2,480	1,264	49%
Group 4	Others	2,508	1,397	44%
Group 5	Country	2,560	1,343	48%
Total		12,573	6,681	47%

Table 11. Effect of the treatments on the attrition rate.

REMINDERS	Attrition
Generic	0.005
	(0.019)
You	0.008
	(0.017)
Family	0.027
	(0.017)
Others	-0.019
	(0.017)
Country	0.011
	(0.017)
Constant	0.450
	(0.304)
Controls	Yes
Observations	12,573

Notes: Attrition between the first and the second survey. Controls include the following balancing covariates (used at the randomisation stage): gender, age, region, education and household income. Robust standard errors in parentheses. Confidence: *** p<0.01, ** p<0.05, * p<0.1.
