# Supplementary material for: Promoting social distancing in a pandemic: Beyond good intentions
Source: PLoS One. 2021 Dec 2;16(12):e0260457. doi: 10.1371/journal.pone.0260457 (PMC8638903; doi:10.1371/journal.pone.0260457)
Supplement: Attachment — Submitted filename: Falco & Zaccagni_Response to Reviewers.pdf [file pone.0260457.s002.pdf]

Dr. Nikolaos Askitas
Academic Editor
PLOS ONE

1 November 2021

Dear Dr. Askitas,

Re: PONE-D-21-25931
"Promoting social distancing in a pandemic: Beyond the good intentions"

Thank you for giving us the opportunity to revise and resubmit our article to PLOS ONE (PONE-D-21-25931). We are grateful for the constructive set of comments that you and the three referees provided. We have been able to address them in full and we believe the paper is now greatly improved as a result.

In this document, we provide a detailed account of our responses to each one of the comments we received. We begin by addressing your own remarks and we continue with the points raised by the referees.

Overall, we believe this revision has helped us to sharpen the paper. We are very grateful for your time in considering our article and for having invited us to resubmit.

Yours sincerely,

Paolo Falco and Sarah Zaccagni

Editor
(Note: We report the referee's comments in italics and our responses in normal font.)

> *I would be happy to receive and evaluate a revision which responds to the comments by the reviewers.*
>
> *Also here is a point which troubles me and I would like a response on. You say in the abstract: "This is despite the possibility that respondents may tend to over-report compliance."*
> *My point is that you feel your results are shielded from over-reporting compliance because of obvious reasons. Let me propose though that the "planning fallacy" could be trouble here (https://en.wikipedia.org/wiki/Planning_fallacy).*

Thank you for this important remark. The reason for making that point in the abstract is that our key result, a gap between intentions and actions, is detected *despite the fact that respondents may over-report compliance*. In other words, the gap we detect is likely to be lower than the actual gap we should find if people reported truthfully. Nonetheless, we find the gap to be sizeable. This point is discussed on page 16 and we decided to remove the above sentence from the abstract for the sake of conciseness. Regarding the planning fallacy, we agree that it should be taken into consideration and we are very grateful for the remark. In our view, this constitutes one of the *channels* that could help to explain our results. Respondents who declare an intention to comply may be unable to foresee that when the time comes complying may prove more difficult than they expected. We believe our results are consistent with this hypothesis and we discuss this in the manuscript (page 16, footnote 20), where we are explicit about the fact that the intention-to-action-gap we detect may be due to different mechanisms, including the planning fallacy.

> *Also you compare reports about things people say they will do "tomorrow" with things they say they did "yesterday" at a later point in time. From Fig. E.1 I suppose "tomorrow" and "yesterday" are different weekdays which opens you to day-of-week effects you need to account for. What happens if you control for day of week in your regression?*

This is a valid concern, thank you. First, we should remark that in order to limit issues of this kind we exclude from the analysis respondents who refer to a weekday in their first interview and to a weekend day in their follow-up interview (and vice versa). We generally find, however, that such mismatches do not play a major role. When we run the analysis on the full sample in a robustness check, we find that our conclusions do not change (Table D.1). Furthermore, following this suggestion, we have included the day of the week among the control variables in our empirical specifications and our results are unchanged. The additional estimates are in Table D.2, Appendix D. We also report them here:

VARIABLES	(1)<br>Intention	(2)<br>Action	(3)<br>Intention	(4)<br>Action
You	0.044***	0.0287		
	(0.016)	(0.0188)		
Family	0.043***	0.0217		
	(0.016)	(0.0189)		
Others	0.011	0.0135		
	(0.016)	(0.0185)		
Country	0.025	0.0297		
	(0.016)	(0.0188)		

You loss			0.0448**	0.0273
			(0.0196)	(0.0223)
You gain			0.0428**	0.0300
			(0.0193)	(0.0221)
Family loss			0.0470**	0.0250
			(0.0198)	(0.0223)
Family gain			0.0398**	0.0183
			(0.0197)	(0.0223)
Others loss			0.00412	-0.000498
			(0.0181)	(0.0214)
Others gain			0.0182	0.0261
			(0.0181)	(0.0216)
Country loss			0.0268	0.0170
			(0.0185)	(0.0215)
Country gain			0.0236	0.0436*
			(0.0189)	(0.0227)
Generic	0.025	0.0321	0.0248	0.0321
	(0.019)	(0.0224)	(0.0190)	(0.0224)
Controls	yes	yes	yes	yes
Observations	5,310	5,310	5,310	5,310
R-squared	0.009	0.006	0.010	0.007

Also how far away from home people will go and how long that will last is affected by weather conditions which might differ across space and time. A comment on why this does not play a role here would appear to be necessary. If it could you might need to add weather in your regression (precipication, temperature, wind speeds should be easy to get).

We are grateful for this remark and we entirely agree with your intuition. Weather conditions can indeed affect the choice to stay home or go out. In order to address such concerns, we used random assignment to treatment and control, which ensures that weather conditions are the same, on average, across groups and are not responsible for the treatment effects we detect. A formal test of balance is provided in Table A.1, where we see that the distribution of respondents across regions (i.e., the share of respondents in each region) is approximately the same in all groups. This is a strong indication that weather conditions, which are likely to differ across regions, were balanced across treatment groups. Since we do not have more precise information on the geolocalisation of respondents, we cannot do better than testing balance at the regional level, but we believe our design is sufficiently strong nonetheless.

Finally something minor. Please add the year in Fig. E.1. Years from now it will make the life of readers easier plus a Fig. should contain all the info necessary to fully understand it.

Thank you for spotting this. We have added the year to the dates.

Referee R1

(Note: We report the referee's comments in italics and our responses in normal font.)

> *While the contribution is not revolutionary, nor the proposed solutions (from the experimental treatments) particularly strong, it is well-described and motivated and contributes to our understanding of how to improve COVID messaging around the world. Therefore I suggest acceptance with a few very minor revisions (some other comments below as well):*
>
> *- maybe there is a difference b/t American and Italian English, but I think grammatically the subtitle would be more correct as "beyond good intentions"*

Thank you for your careful reading of our paper. We agree with this comment and we have changed the title accordingly.

> *- I would change the first line of the abstract to "Do reminders to promote social distancing achieve desired effects in behavior, not just intentions?" to highlight your contribution better*

We are grateful for the suggestion and we agree. We are slightly concerned, however, that readers may find the new sentence to be at odds with the fact that we investigate self-reported behaviour (which we explain later in the abstract). For this reason, we have chosen to keep our more general formulation, but we would be happy to revise it if the referee has a strong preference in this regard.

> *- nice experimental set-up with impressive sample size and compliance*

Thank you for your positive remarks.

> *- it wasn't totally clear from the main text that your validation with the apple data was aggregate and not individual-level; written right now to suggest it is individual-level -- please clarify this*

This is a very good point, thank you. We have clarified the text to explain that we use aggregate data from Apple. The test we propose is nonetheless robust.

> *- table 1: in my mind, the column names for frame and domain should be switched; e.g. "family" is a domain*

Thank you for this comment. We agree and we have implemented the suggestion.

> *- I found the robustness checks generally convincing*

Once again, thank you for your positive feedback.

<u>Referee R2</u>

(Note: We report the referee's comments in italics and our responses in normal font.)

> *The manuscript details the results of an experiment of whether reminding people to comply with public health guidelines impacts their intentions and actual behaviors in the context of COVID-19. I think the results are interesting, novel, and timely.*
>
> *My only real concern is that there is almost no theory to explain these results. I think the authors need to work to provide a theoretical foundation for their findings. Certainly, there is a significant amount of theory to work with from micro-economics and psychology on the efficacy of nudging experiments, and/or the utility of how narratives are framed.*

Thank you for this important remark. We have reflected carefully on this point and the revised manuscript contains a new section that outlines a simple theoretical framework to guide the reader's thinking (Section 3: Theoretical Framework). The main idea is that reminders act as a nudge that helps respondents to make more *attentive* choices. The model clarifies that we do not believe the nudges alter people's preferences or information sets, they simply increase their attentiveness and make the relative utility of different choices more salient. In the specific case at hand, people may be inattentive to the fact that going out can have detrimental impacts on their own health and on the health of others (indeed, the stylised model accounts for both own utility and other people's welfare). The nudge reminds them of that. We believe this is a better characterisation than assuming the reminders provided *new* information, given that basic knowledge about COVID-19 spread very quickly at the onset of the pandemic. In light of this simple model, the fact that we do not detect strong impacts of the reminders on behaviour is consistent with the fact that COVID-19 became a salient issue very rapidly, leaving limited scope for our messages to increase attentiveness. Nonetheless, we make an important contribution by documenting a sizeable gap between intentions and actions.

Referee R3

(Note: We report the referee's comments in italics and our responses in normal font.)

> *This manuscript uses a messaging intervention to examine how to increase compliance with stay at home or social distancing behavior in Demark. The authors find that the message influences behavioral intention in the expected direction, but does not influence longer term behaviors. Overall, it's a good paper – the study is competently designed, and the results are described accurately, so I'm happy to recommend relatively minor revisions.*
>
> *One thing that might be nice to see is hypotheses delineated in the text more clearly. It's obvious the authors have them (it's an experiment and its pre-registered!), but it's not really clear in text what they are before we get to the research design.*

Thank you for this suggestion. We agree that laying out our hypotheses more explicitly earlier in the paper can enhance clarity. We have done so in the revised manuscript (page 3).

> *I found the discussion of prospect theory a bit lacking. The discussion of the literature on COVID-19 and public health measures is good, but prospect theory gets only kind of a passing citation to a 2013 review article. I'd like to see more here, especially since PT is so relevant to the design of the study (though, perhaps not to the analyses).*

We are grateful for this remark. We now refer to prospect theory earlier in the manuscript (page 3), when we outline the hypotheses being tested (see our response to the previous comment). We have also expanded the discussion of prospect theory in the Experimental Design section (page 5-6) along the lines of the reflections outlined in the response to the next comment.

> *This brings me to a point that the authors can't avoid, but one that I think merits discussion. The authors generally find no differences (either in intention or action) based on the loss vs. gain framing. Obviously, the authors are aware that PT predicts differences in risk seeking behavior on losses vs. gains – risk seeking in domain of losses, and risk aversion in the domain of gains. I would guess this translates to – more likely to stay home in gain frame, less likely in the loss frame.*
>
> *Of course, I can see some explanations of why this might be the case. It doesn't totally fit the PT framework – the risk individuals take by leaving home doesn't seem to necessarily have a payoff with respect to public health outcomes. This is a divergence from the classic PT public health work, where the "risky' option has direct positive public health consequences if the risk pays off. So, I see this less as a limitation to the work and more as a limitation to PT generally – if there are no clear and relevant benefits to the risk, loss/gain framing seems to have no impact on behaviors. I think this is definitely worth discussing, as it expands this manuscript from speaking to COVID-19/public health literature to speaking generally to PT and decision-making literature.*

This is a great comment, thank you, and it deserves some discussion. As you point out, prospect theory predicts that people should be more willing to make a risky health choice when the alternative is framed in the loss domain than when it is framed in the gain domain. The classic example in this area comes from the seminal work of Kahneman and Tversky. In a famous experiment, they presented respondents with a hypothetical scenario in which a new disease is sweeping across the country and scientists must choose between two programmes. Programme A can save 200 lives, Programme B is a risky lottery with a one-third probability that 600 people

will be saved and a two-thirds probability that nobody will be saved. They found that when the same two programmes are framed in the loss domain (Programme A: 400 people will die; Programme B: one-third probability that nobody will die and two-thirds probability that 600 people will die), respondents are more likely to choose the risky option. We are, however, in a different situation, as correctly pointed out by the referee. In our case, we can interpret going out as the risky choice, which has potentially *detrimental* effects (rather than beneficial ones, as in the case of the medical programmes above). When we frame such a choice in the loss domain, therefore, we should expect to trigger the opposite reaction to the example above. In other words, framing our message in the loss domain should trigger lower risk taking, since we are looking at a downside risk (becoming infected with COVID-19) rather than an upside risk (finding a way to cure a disease).

Prospect theory also makes another closely related prediction that is more directly applicable in our context. According to the theory, losses motivate behaviour more than equivalent gains. This is because in prospect theory decision making is based on *value relative to a reference point* and people typically weigh losses more than equivalent gains. The theory has found confirmation across a range of contexts. A classic example related to the scenario above is that medical treatments described as having a "75% survival rate" are viewed more positively than those with a "25% mortality rate", even though they are de facto equivalent (Levin, Schnittjer, & Thee, 1988; Marteau, 1989; Wilson, Kaplan, & Schneidermann, 1987). It follows that people are more responsive to incentives framed as potential losses relative to an initial endowment than to equivalent gains relative to a reference point of 0. Recent work demonstrates this in the context of a smoking-cessation intervention where incentives framed as losses relative to an endowment had significantly stronger effects than incentives framed as equivalent gains relative to no initial endowment (Romanowich, & Lamb, 2013). A vast literature offers similar examples in different domains (Hossain, & List, 2012, Fryer, Levitt, List, & Sadoff, 2012, Imas, Sadoff, & Samek, 2017). Our work builds directly on this literature and investigates framing effects in the context of an intervention to promote social distancing. The reason we find little evidence of such framing effects may be that the differences between our alternative framings are quite subtle. Some respondents are reminded that going out may lead them to become infected (a loss). Some other respondents are reminded that staying home would prevent them from getting infected (a gain, but only relative to a hypothetical situation in which going out leads to becoming infected). Since we were interested in identifying the most effective messages to inform policymaking at the height of the pandemic, testing these differences was valuable, but it is not unreasonable to hypothesise that the variations might have been too subtle to trigger the behavioural patterns predicted by prospect theory.

We now provide a clearer discussion of these issues in the manuscript (Page 5-6, and page 13). We are grateful to the referee for giving us the opportunity to reflect on these important issues.

> *Another point worth making is that the differences between intentions and actions themselves are possibly not statistically different (though eyeballing it looks like it might be close!). This doesn't change the quality of the authors' work, but does probably change their interpretation a bit. I'm hesitant to say there are no behavioral effects here – all are in the expected direction, save 1 which is functionally zero, and all may have reasonable effect sizes (excuse the gross reference to my own work, but a paper from Roberts and Utych shows generally small effects on social distancing behavioral differences in the US - https://www.researchgate.net/publication/348607480_Polarized_Social_Distancing_Residents_*

of_Republican-Majority_Counties_Spend_More_Time_Away_from_Home_During_the_COVID-19_Crisis). The authors have quite a few observations, but they also have quite a few groups. They're still at roughly 590 per group, which should be fine for detecting effects, but I'd be more comfortable saying the effect sizes get smaller, rather than disappear, given that these probably are small effects that would be distinguished in a very large sample (as is available with some behavioral data out there).

This is a great remark and we entirely agree, thank you. Section 6.1 offers a discussion of the fact that finding no behavioural change may result from limited statistical power, which makes it difficult to detect effects that are likely to be small. A more prudent conclusion, therefore, is that behavioural impacts are *smaller* than impacts on intentions. We have made this clearer in the draft.

Overall, I hope I have conveyed that I have a positive assessment here, and that the authors are doing really good and well-designed work. I think this should be published, pending these small revisions.

We are grateful for the positive feedback and constructive comments.

References:

Fryer, R. G., Levitt, S. D., List, J., & Sadoff, S. (2012). *Enhancing the efficacy of teacher incentives through loss aversion: A field experiment* (No. w18237). National Bureau of Economic Research.

Hossain, T., & List, J. A. (2012). The behavioralist visits the factory: Increasing productivity using simple framing manipulations. *Management Science*, *58*(12), 2151-2167.

Imas, A., Sadoff, S., & Samek, A. (2017). Do people anticipate loss aversion?. *Management Science*, *63*(5), 1271-1284.

Levin, I. P., Schnittjer, S. K., & Thee, S. L. (1988). Information framing effects in social and personal decisions. *Journal of experimental social psychology*, *24*(6), 520-529.

Marteau, T. M. (1989). Framing of information: Its influence upon decisions of doctors and patients. *British Journal of Social Psychology*, *28*(1), 89-94.

Romanowich, P., & Lamb, R. J. (2013). The effect of framing incentives as either losses or gains with contingency management for smoking cessation. *Addictive behaviors*, *38*(4), 2084-2088.

Wilson, D. K., Kaplan, R. M., & Schneiderman, L. J. (1987). Framing of decisions and selections of alternatives in health care. *Social Behaviour*, *2*(1), 51-59.
